# Acetylated and Methylated β-Cyclodextrins as Viable Soluble Supports for the Synthesis of Short 2′-Oligodeoxyribo-nucleotides in Solution

**DOI:** 10.3390/molecules171012102

**Published:** 2012-10-16

**Authors:** Alejandro Gimenez Molina, Vyacheslav Kungurtsev, Pasi Virta, Harri Lönnberg

**Affiliations:** Department of Chemistry, University of Turku, FIN-20014 Turku, Finland; Email: algimo@utu.fi (A.G.M.); vyakun@utu.fi (V.K.); pamavi@utu.fi (P.V.)

**Keywords:** cyclodextrin, oligonucleotides, phosphoramidites, soluble support, synthesis

## Abstract

Novel soluble supports for oligonucleotide synthesis **11a**–**c** have been prepared by immobilizing a 5′-*O*-protected 3′-*O*-(hex-5-ynoyl)thymidine (**6** or **7**) to peracetylated or permethylated 6-deoxy-6-azido-β-cyclodextrins **10a** or **10b** by Cu(I)-promoted 1,3-dipolar cycloaddition. The applicability of the supports to oligonucleotide synthesis by the phosphoramidite strategy has been demonstrated by assembling a 3′-TTT-5′ trimer from commercially available 5′-*O*-(4,4′-dimethoxytrityl)thymidine 3′-phosphoramidite. To simplify the coupling cycle, the 5′-*O*-(4,4′-dimethoxytrityl) protecting group has been replaced with an acetal that upon acidolytic removal yields volatile products. For this purpose, 5′-*O*-(1-methoxy-1-methylethyl)-protected 3′-(2-cyanoethyl-*N*,*N*-diisopropyl-phosphoramidite)s of thymidine (**5a**), *N*^4^-benzoyl-2′-deoxycytidine (**5b**) and *N*^6^-benzoyl-2′-deoxyadenosine (**5c**) have been synthesized and utilized in synthesis of a pentameric oligonucleotide 3′-TTCAT-5′ on the permethylated cyclodextrin support **11c**.

## 1. Introduction

The methods currently applied to preparation of oligonucleotides in the laboratory [1,2] as well as in large-scale production [3,4,5], consist of stepwise addition of nucleoside phosphoramidites to a growing oligomer chain on a solid support. While solid-supported phosphoramidite chemistry undoubtedly is the method of choice for small-scale synthesis of oligonucleotides, solution phase synthesis may well challenge it in cases where multikilogram quantities are needed for clinical phase trials of therapeutic oligonucleotides or as starting materials for the construction of nanomaterials. To ensure efficient coupling on a solid support, the expensive phosphoramidite monomers have to be used in large excess and this requirement tends to become even more stringent in scaling up the synthesis [6]. In addition, the support itself is expensive, comprising one third of the overall raw material cost [4]. For these reasons, several strategies for the assembly of oligonucleotides on soluble supports have been suggested. Most of these are based on use of polyethylene glycol (PEG) as a support. PEG allows coupling in MeCN and may be precipitated by Et_2_O. This protocol has been applied to the synthesis of both oligonucleotides and their phosphoromonothioate analogs by the phosphoramidite [7,8,9,10,11], *H*-phosphonate [12], and phosphotriester [13,14] strategies. More recently, the 3′-*O*-(adamantan-1-yl)acetyl group [15] and 3′-*O*-succinyl-tethered 1-ethyl-3-methylimidazolium tetrafluoroborate salt [16] have been used as soluble supports. The (adamantan-1-yl)acetyl group allows extractive work-up of the growing oligonucleotide chain that, besides conventional benzoyl and isobutyryl protections, additionally bears a pivaloyloxymethyl group at *N*3 of the thymidine residues [15]. The imidazolium tetrafluoroborate tagged oligomers have, in turn, been separated from small molecular reagents by successive precipitations and extractions. As a complementary approach, solid-supported reagents have been exploited to synthesize oligonucleotides in solution [17,18].

We now report on an alternative approach based on the use of fully protected β-cyclodextrin as a soluble support and replacement of the conventional 5′-*O*-(4,4′-dimethoxytrityl) protecting group with an acetal that upon removal gives only volatile products. The cyclodextrin support is sufficiently small to allow efficient coupling and accurate mass spectrometric analysis, but it still is hydrophobic enough so that the separation of the growing oligonucleotide chain from all the reagents can be rapidly realized by flash chromatography with limited solvent consumption. On using methylated cyclodextrin, the support may be removed by extraction after the ammonolytic release and deprotection of the oligonucleotide.

## 2. Results and Discussion

### 2.1. Preparation of Nucleosidic Building Blocks

**Scheme 1:**
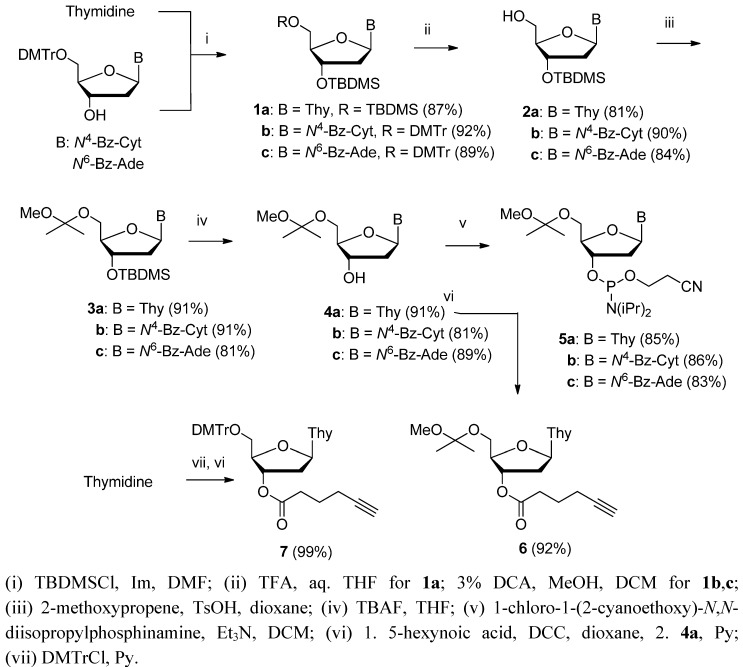
Preparation of nucleosidic building blocks.

Scheme 1 outlines the preparation of 5′-*O*-(1-methoxy-1-methylethyl) protected 2′-deoxy-ribonucleoside 3′-phosphoramidites used as building blocks in the oligonucleotide chain assembly. The 3′-*O-*(*tert*-butyldimethylsilyl)ated nucleosides **2a**–**c**, used as the starting materials for the introduction of the 5′-acetal protection, were obtained in two different manners. Thymidine was first converted to 3′,5′-di-*O*-(*tert*-butyldimethylsilyl)thymidine (**1a**) and the 5′-*O*-silyl group was then removed by acid-catalyzed hydrolysis to obtain **2a**. Commercially available *N*-benzoylated 5′-*O*-(4,4′-dimethoxytrityl)-2′-deoxy-cytidine and -adenosine were, in turn, converted to their 3′-*O*-(*tert*-butyldimethylsilyl) counterparts by silylation of **1b**,**c** and subsequent acid-catalyzed detritylation to **2a**,**c**. The exposed 5′-hydroxy function was then subjected to acid-catalyzed acetalization with 2-methoxypropene to obtain 5′-protected nucleosides **3a**–**c**, which were desilylated to afford **4a**–**c**.

The phosphoramidite building blocks **5a**–**c** were finally obtained by phosphitylating the exposed 3′-hydroxy function with 1-chloro-1-(2-cyanoethoxy)-*N*,*N*-diisopropylphosphinamine. To enable immobilization to an azido-functionalized support, the acetal protected thymidine **4a** was esterified with 5-hexynoic acid to obtain **6**. For comparative purposes, a similar derivative **7** was prepared from 5′-*O*-(4,4′-dimethoxytrityl)thymidine.

### 2.2. Preparation of Cyclodextrin-Derived Supports

**Scheme 2:**
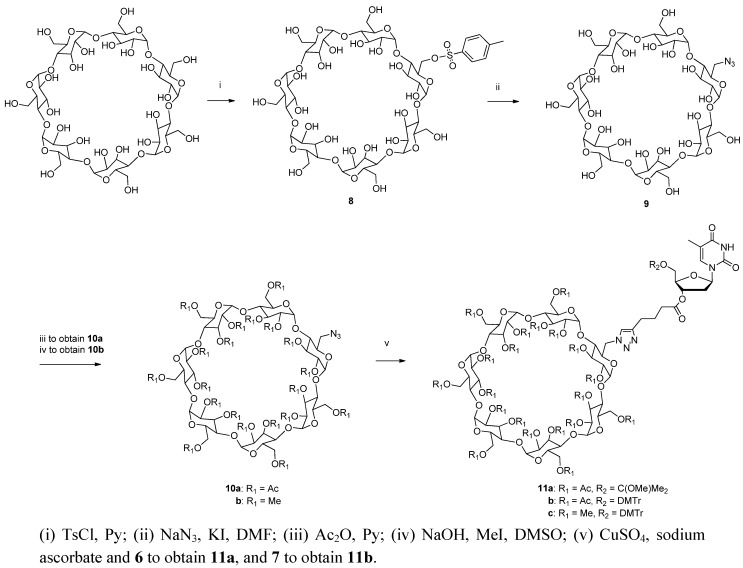
Preparation of cyclodextrin supports.

**Figure 1:**
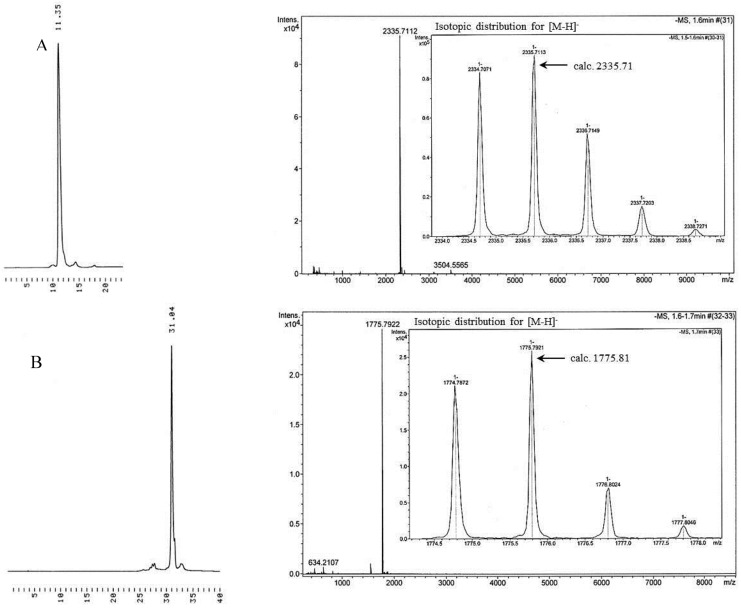
Negative ion ESI-MS ([M−H]^−^) and HPLC traces for the thymidine derivatized cyclodextrin supports **11a** (**A**) and **11c** (**B**) after removal of the 5′-*O*-protecting group from thymidine. Chromatographic conditions: a Thermo ODS Hypersil C18 (250 × 4.6 mm, 5 µm) column eluted with a mixture of MeCN and aq. triethylammonium acetate (0.1 mol·L^−1^) at flow rate 1 mL·min^−1^. For support A, a linear gradient elution from MeCN 50% at *t* = 0 to MeCN 100% at *t* = 20 min. For support B, a linear gradient from MeCN 0% at *t* = 0 to MeCN 60% at *t* = 25 min, and then to MeCN 100% at *t* = 30 min.

The soluble supports utilized in the oligonucleotide chain assembly were prepared by immobilizing 5′-protected 3′-*O*-(hex-5-ynoyl)thymidines **6** or **7** on an azido-functionalized peracetylated or permethylated β-cyclodextrin (Scheme 2). For this purpose, β-cyclodextrin was first monotosylated to **8** [19] and the tosyl group was displaced with azide ion. The azido-functionalized cyclodextrin **9** obtained was then acetylated with excess of acetic anhydride in pyridine, yielding **10a**, or methylated to **10b** with an equimolar mixture of sodium hydroxide and methyl iodide in DMSO [20,21]. The alkyne-functionalized thymidines, either **6** or **7**, were finally conjugated to the azido group by Cu(I) promoted 1,3-dipolar cycloaddition [22,23]. The identity and homogeneity of the products before and after conjugation were checked by ESI-MS and HPLC. The data referring to acetylated and methylated supports **11a** and **11c** after removal of the 5′-*O*-protecting group from thymidine is given in Figure 1.

### 2.3. Assembly of Oligonucleotides from 5′-O-(4,4′-Dimethoxytrityl) Building Blocks

**Figure 2:**
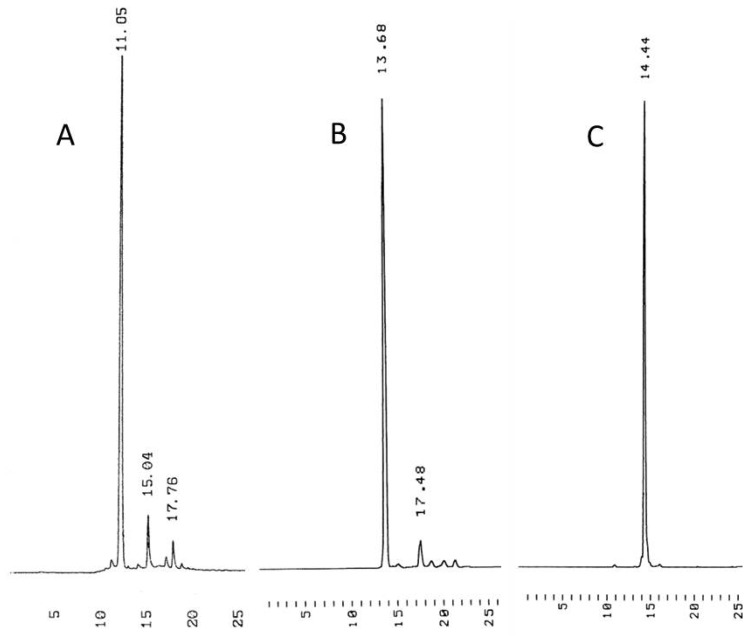
HPLC traces of the crude product of 3′-TTT-5′ prepared from 5′-*O*-(4,4′-dimethoxytrityl)thymidine phosphoramidite on acetylated cyclodextrin support **11b** (traces **A**), from 5′-*O*-(1-methoxy-1-methylethyl)thymidine phosphoramidite (**5a**) on acetylated support **11a** (traces **B**), and from the same building block (**5a**) on methylated support **11c** (traces **C**). Chromatographic conditions: a Thermo ODS Hypersil C18 (250 × 4.6 mm, 5 µm) column eluted with a mixture of MeCN and aq. triethylammonium acetate (0.1 mol·L^−1^) at flow rate 1 mL·min^−1^. A linear gradient from MeCN 0% at *t* = 0 to MeCN 50% at *t* = 20 min, and then to MeCN 100% at *t* = 25 min.

The efficiency of phosphoramidite coupling on a cyclodextrin support was first tested by using commercially available 5′-(4,4′-dimethoxytrityl)thymidine 3′-(2-cyanoethyl-*N*,*N*-diisopropyl-phosphoramidite) building blocks for coupling on the peracetylated support **11b**. Accordingly, the DMTr group of **11C** was removed by 1 h treatment with 3% dichloroacetic acid in DCM, a DCM/HCO_3_^−^-workup was carried out and the organic phase was evaporated to dryness. Phosphoramidite coupling was then carried out in MeCN (1 h at r.t.), using 1.5 equiv. of the phosphoramidite building block and tetrazole compared to support-bound thymidine. The coupling cycle was completed by conventional I_2_ oxidation in aqueous lutidine/THF and subsequent DCM/HSO_3_^−^-workup. After the second similar coupling step, the overall yield of the support bound fully protected thymidine trimer determined by UV-spectroscopy was 85%, corresponding to 92% coupling yield. The homogeneity of the product was verified by RP-HPLC after ammonolytic release from the support (HPLC trace A in Figure 2). 

### 2.4. Assembly of Oligonucleotides from 5′-O-(1-Methoxy-1-methylethyl) Building Blocks

**Figure 3:**
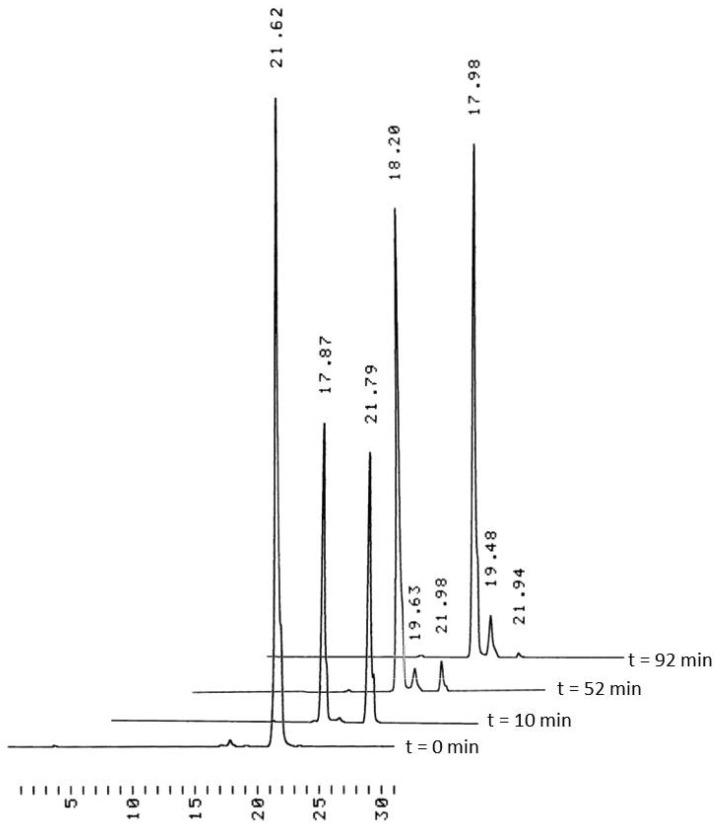
HPLC traces of *N*^6^-benzoyl-5′-*O*-(1-methoxy-1-methylethyl)-2′-deoxyadenosine (2 mmol·L^−1^) treated for 10 min with HCl (10^−4^ mol·L^−1^) in a 2:1 (*v/v*) mixture of 1,4-dioxane and MeOH at 25 °C. Notation: Starting material at 21.6–22.0 min, *N*^6^-benzoyl-2′-deoxyadenosine at 17.8–18.2 min and *N*^6^-benzoyladenine at 18.8–19.6 min. Chromatographic conditions: a Thermo ODS Hypersil C18 (250 × 4.6 mm, 5 µm) column eluted with a mixture of MeCN and aq. triethylammonium acetate (0.1 mol·L^−1^) at flow rate 1 mL·min^−1^. A linear gradient from MeCN 0% at *t* = 0 to MeCN 60% at *t* = 25 min, and then to MeCN 100% at *t* = 30 min.

Although the initial studies described above clearly show that high coupling yield may be achieved on a cyclodextrin support even when using only 50% excess of the monomeric building block, the protocol utilized do not allow synthesis of longer sequences, owing to accumulation of 4,4′-dimethoxytrityl alcohol upon repeated couplings. Removal of this compound from the reaction mixture prior to next coupling is difficult, since the support bearing the protected oligonucleotide and dimethoxytrityl alcohol both are rather hydrophobic and, hence, removal by extraction or flash chromatography is not straightforward. For this reason, it appeared attractive to replace the conventional dimethoxytrityl protection with an acid-labile acetal protection, *viz*. with 1-methoxy-1-methylethyl group, which upon acid-catalyzed transesterification with MeOH was released as volatile dimethyl acetal of acetone. As seen from Figure 3, removal of the 5′-protecting group by HCl in a 2:1 (*v/v*) mixture of 1,4-dioxane and MeOH was sufficiently faster than depurination of N6-benzoyl-2′-deoxyadenosine to allow virtually quantitative removal of the acetal protection without appreciable depurination.

The synthesis of 3′-TTT-5′ was then repeated using **11a** as a support and **5a** as a building block. The 5′-*O*-(1-methoxy-1-methylethyl) protection was first removed by 30 min treatment with HCl (0.1 mol·L^−1^) in a 2:1 mixture of dioxane and MeOH. The volatiles were removed under reduced pressure and traces of MeOH were removed by coevaporation with dry pyridine. It is worth noting that traces of pyridine in the product do not harm the subsequent coupling. ESI^-^-MS analysis of the residue verified quantitative removal of the protecting group; only the [M−H]^−^ signal at *m/z* 2335.71 referring to the deprotected product appeared, no sign of the [M−H]^−^ signal of 2407.75 corresponding to the protected support was observed.

Deprotected **11a** was then subjected to coupling with 1.5 equiv. of **5a** in MeCN under nitrogen, using 4,5-dicyanoimidazole as an activator (6 h at r.t.), and the phosphite triester obtained was oxidized by addition of I_2_ in aq. lutidine/THF (30 min at r.t.). Upon DCM/HSO_3_^−^-workup, lutidine was partly transferred to the organic phase and, hence, the soluble-supported product obtained by evaporation of DCM still contained traces of lutidine. The residue was dissolved in a 2:1 mixture of dioxane and MeOH and the pH of the solution was adjusted between 4 and 5 to remove the 5′-acetal protection and the reaction was allowed to proceed for 1 h. To remove the traces of reagents and the products formed from the unreacted building block upon oxidation, the support was purified by flash chromatography on a short silica gel column before the next coupling. The overall yield after two couplings, determined by UV-spectroscopy, was 87%, corresponding to 93% coupling yield. The trimer released by ammonolysis was homogeneous by RP-HPLC (trace B in Figure 2).

The shortcoming of acetylated cyclodextrin as a support is that it does not withstand ammonolysis, but is converted to hydrophilic products that render the isolation of the oligonucleotide product difficult, necessitating reversed phase HPLC purification. To avoid this, the acetylated cyclodextrin support **10a** was replaced with the methylated one, **11c**, and the synthesis of 3′-TTT-5′ was repeated. The yield of the synthesis and the homogeneity of the product were similar to those observed for the acetylated support, but now the support could be removed by extraction after the ammonolysis. The HPLC traces of the crude product mixture are shown in Figure 2C.

**Figure 4:**
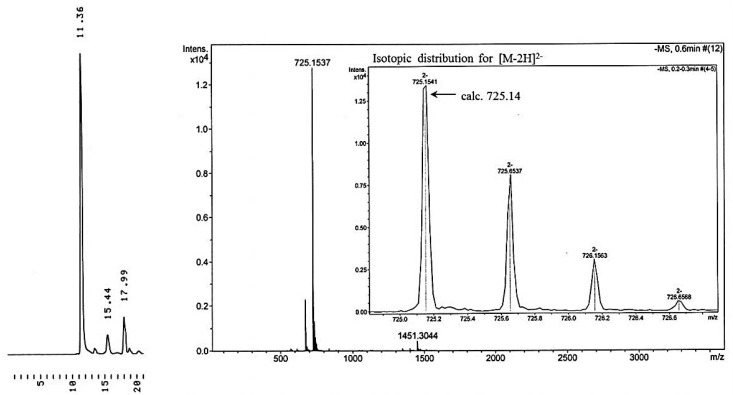
HPLC traces and ESI-MS of the crude product of 3′-TTCAT-5′. For the chromatographic conditions, see the legend of Figure 2

Finally a hetero-sequence containing 2′-deoxyadenosine and 2′-deoxycytidine in addition to thymidine was assembled on support **11c**. Accordingly, the deprotected support bearing the first nuclesoide was dissolved in MeCN and 1.5 equiv. of the monomeric building block in MeCN was added dropwise, followed by 1.5 equiv. of 4,5-dicyanoimidazole in MeCN. The final concentration of **5a** in the reaction mixture was 40 mmol L^−1^. The reaction was allowed to proceed for 20 h at r.t. after which oxidation with iodine in a 6:1 (*v/v*) mixture of aq. THF and 2,6-lutidine was carried out (45 min). After aq. NaHSO_3_/DCM work-up, the organic phase was dried over Na_2_SO_4_, filtered and evaporated to dryness under reduced pressure. The residue was dissolved in a 2:1 (*v/v*) mixture of dioxane and MeOH and HCl in dioxane (0.1 mol·L^−1^) was added to adjust the pH to 4. After 2 h agitation, the mixture was evaporated to dryness and coevaporated with pyridine and MeCN. A rapid column chromatography on a short column was applied to remove the excess of the unreacted building block. Fractions containing the desired product were mixed, evaporated by under reduced pressure and dried under high vacuum. The identity of the support bound dimer was verified by ESI-MS. The same coupling cycle was then repeated by using **5b**, **5c** and again **5a** as building blocks. However, the coupling mixture contained about 20% DCM to ensure complete dissolution of the cyclodextrin-bound starting material. Finally, conventional ammonolysis was carried out to release and deprotect the pentameric oligonucleotide and the support was removed by equilibration between water and DCM. The gravimetrically determined overall yield of the pentamer was 52%, corresponding to 85% average coupling yield. Figure 4 shows the identity and homogeneity of the pentamer. 

## 3. Experimental

### 3.1. General

Reactions were monitored by TLC (Merck, Silica gel 60 F_254_), using short wavelength UV, KMnO_4_ staining or burning with 10% aq. H_2_SO_4_ for detection. 1D and 2D NMR spectra were recorded on a Bruker Avence 500 MHz or 400 MHz at 25 °C. The chemical shifts are given in ppm. Mass spectra were recorded on a Bruker Daltonics MicrOTOF-Q spectrometer using ESI ionization. RP HPLC was performed on a Thermo ODS Hypersil C18 (250 × 4.6 mm, 5µm) column using UV detection at 260 nm.

### 3.2. Materials

*3′,5′-Di-O-(*tert*-butyldimetylsilyl)thymidine* (**1a**) was synthesized as described previously [24]. Thymidine (41.3 mmol; 10.02 g) was dissolved in DMF (35 mL). Imidazole (264 mmol, 18.0 g) in DMF (65 mL) and *tert*-butyldimethylsilyl chloride (131 mmol; 19.87 g) in DMF (25 mL) were added. The mixture was stirred at r.t. for 70 h and then equilibrated between water (50 mL) and diethyl ether (50 mL). The organic phase was washed twice with water, dried over Na_2_SO_4_ and concentrated to white solid (87%, 16.93 g). The crude product was subjected to desilylation (*cf.* preparation of compd. **2a**) without further purification. The ^1^H- and ^13^C-NMR spectra were identical to those reported in literature [25,26]. Positive ion ESI-MS: *m/z* obsd. 471.28 [M+H]^+^, 493.27 [M+Na]^+^, 509.24 [M+K]^+^; calcd. 471.27 [M+H]^+^, 493.25 [M+Na]^+^, 509.23 [M+K]^+^.

*N^4^-Benzoyl-5′-O-(4,4′-dimethoxytrityl)-3′-O-(tert-butyldimethylsilyl)-2′-deoxycytidine* (**1b**). Commercially available *N*^6^-benzoyl-5′-*O*-(4,4′-dimethoxytrityl)-2′-deoxycytidine (1.42 mmol, 0.90 g) was dissolved in DMF (5 mL). *tert*-Butyldimethylsilyl chloride (0.33 g, 2.35 mmol, 1.5 equiv.) and imidazole (0.29 g, 4.70 mmol, 3 equiv.) were added and the reaction was allowed to proceed for 15 h at r.t. The mixture was equilibrated between water and EtOAc. The organic phase was dried on Na_2_SO_4_ and concentrated to dryness, giving **1b** in 92% yield (1.31 mmol, 0.98 g). The ^1^H- and ^13^C-NMR spectra were identical with those reported in literature [27]. Negative ion ESI-MS: *m/z* obsd. 746.32 [M−H]^−^; calcd. 746.33 [M−H]^−^.

*N^6^-Benzoyl-5′-O-(4,4′-dimethoxytrityl)-3′-O-(tert*-*butyldimethylsilyl)-2′-deoxyadenosine* (**1c**) was obtained in 89% yield (1.55 mmol, 1.20 g) from commercial *N*^6^-benzoyl-5′-*O*-(4,4′-dimethoxytrityl)-2′-deoxyadenosine as described above for compd. **1b**. ^1^H-NMR (500 MHz, CDCl_3_): δ = 0.00 (s, 6H, SiMe_2_), 0.84 (s, 9H, SiC*Me*_3_), 2.24–2.29 (m, 1H, H2′), 2.35–2.44 (m, 1H, H2′), 3.18 (d, *J* = 11.2 Hz, 1H, H5′), 3.29 (d, *J* = 11.2 Hz, 1H, H5′), 3.68 (s, 6H, OMe), 3.96–4.00 (m, 1H, H4′), 4.71–4.77 (m, 1H, H3′), 6.38 (m, 1H, H1′), 6.74 (d, *J* = 9.7 Hz, 4H, DMTr), 7.20–7.40 (m, 9H, DMTr), 7.60–8.00 (m, 5H, Bz), 8.24 (s, 1H, H2), 8.56 (s, 1H, H8), 9.52 (s, 1H, NH). ^13^C-NMR (126 MHz, CDCl_3_) δ = −4.6 (SiMe), −3.9 (SiMe), 17.3 (Si*C*Me_3_), 24.8 (SiC*Me*_3_), 38.8 (C2′), 54.6 (OMe), 62.9 (C5′), 71.7 (C3′), 84.1 (C4′), 85.7 (DMTr), 86.0 (C1′), 112.7 (DMTr), 116.9 (DMTr), 126.4 (C5), 127.4 (DMTr), 127.6 (Bz), 127.8 (DMTr), 128.3 (Bz), 129.6 (DMTr), 132.1 (Bz), 133.6 (Bz), 135.3 (DMTr), 142.4 (C8), 144.7 (DMTr), 149.5 (C4), 151.4 (C2), 151.5 (C6), 158.3 (DMTr), 164. 9 (C=O). Negative ion ESI-MS: *m/z* obsd. 770.3509 [M−H]^−^; calcd. 770.3374 [M−H]^−^.

*3′-O-(tert-Butyldimethylsilyl)thymidine* (**2a**). A 1:1:4 (*v/v/v*) mixture of TFA, water and THF (30 mL) was added dropwise on an ice-bath to compd. **1a** dissolved in a minimal volume of THF. After 1.5 h, another portion (30 mL) of the same mixture was added. The progress of desilylation was monitored by TLC and the reaction was quenched after 5.5 h by equilibration between diethyl ether and aq. NaHCO_3_ (sat). The organic phase was washed with aq. NaHCO_3_, dried over Na_2_SO_4_ and concentrated to white solid foam (81%, 10.38 g). The ^1^H-NMR spectrum (500 MHz, CDCl_3_) was identical to that reported previously [28]. ^13^C-NMR (126 MHz, CDCl_3_): δ = −4.9 (SiMe), −4.8 (SiMe), 12.4 (C5-Me), 17.9 (Si*C*Me_3_), 25.7 (SiC(*C*H_3_)_3_), 40.7 (C2′), 61.8 (C5′), 71.9 (C3′), 86.2 (C4′), 87.8 (C1′), 110.8 (C5), 137.0 (C6), 150.6 (C2), 164.2 (C4). Negative ion ESI-MS: *m/z* obsd. 355.15 [M−H]^−^, 391.13 [M+Cl]^−^, calcd. 355.1689 [M−H]^−^, 391.15 [M+Cl]^−^.

*N^4^-Benzoyl-3′-O-(tert-butyldimethylsilyl)-2′-deoxycytidine* (**2b**). Compd. **1b** (1.31 mmol, 0.98 g) was dissolved in DCM (5 mL) containing 3% dichloroacetic acid (150 μL) and MeOH (2 mL) was added. After 2 h stirring at r.t., DCM/aq. NaHCO_3_ work-up was carried out and the organic phase was dried over Na_2_SO_4_. The solvent was removed by evaporation, and the residue was purified by a silica gel column chromatography (a stepwise gradient of 1–10% MeOH in DCM) to obtain **2b** in 90% yield as white solid foam (1.17 mmol, 0.52 g). The ^1^H- and ^13^C-NMR spectra were identical with those reported in literature [19]. Negative ion ESI-MS: *m/z* obsd. 444.20 [M−H]^−^; calcd. 444.20 [M−H]^−^. 

*N^6^-Benzoyl-3′-O-(tert-butyldimethylsilyl)-2′-deoxyadenosine* (**2c**) was obtained in 84% yield (1.32 mmol, 0.62 g) from **1c** as described above for **2b**. The ^1^H-NMR spectrum was identical with that reported in literature [29]. ^13^C-NMR (126 MHz, CD_3_CN): δ = −4.9 (Si*Me*_2_), 17.3 (Si-*C*Me_3_), 24.8 (Si-C*Me*_3_), 40.0 (C2′), 61.6 (C5′), 72.5 (C3′), 85.2 (C4′), 88.7 (C1′), 124.3 (C5), 127.8 (Bz), 128.2 (Bz), 132.2 (Bz), 133.6 (Bz), 142.6 (C8), 149.6 (C4), 151.1 (C2&C6), 165.0 (Bz). High resolution negative ion ESI-MS: *m/z* obsd. 468.2067 [M−H]^−^; calcd. 468.2067 [M−H]^−^.

*5′-O-(1-Methoxy-1-methylethyl)-3′-O-(*tert*-butyldimethylsilyl)thymidine* (**3a**). Compound **2a** (0.42 mmol, 150 mg) was dissolved in 1,4-dioxane (5 mL) and 11 equiv. of 2-methoxypropene (4.47 mmol, 0.400 mL) and 0.02 equiv. of *p*-toluenesulfonic acid monohydrate (4.2 mmol, 0.80 g) in dioxane (4 mL) were added. In 1 h, all the starting material had disappeared. The crude product was purified by silica gel column chromatography using EtOAc containing 1% petroleum ether and Et_3_N as an eluent. The yield was 91%. ^1^H-NMR (500 MHz, CDCl_3_): δ = −0.01 (s, 6H, Si*Me*_2_), 0.81 (s, 9H, SiC*Me*_3_), 1.31 (s, 6H, OC(OMe)*Me*_2_), 1.84 (s, 3H, C5-Me), 1.95–2.06 (m, 1H, H2′), 2.15–2.25 (m, 1H, H2′), 3.14 (s, 3H, OC(O*Me*)Me_2_), 3.47–3.92 (m, 3H, H4′&2 × H5′), 4.32–4.36 (m, 1H, H3′), 6.24–6.26 (m, 1H, H1′), 7.46 (s, 1H, H6), 10.00 (s, 1H, NH); ^13^C NMR (126 MHz, CDCl_3_): δ = −4.9 and −4.8 (Si*Me*_2_), 12.4 (C5-Me), 17.9 (Si-*C*Me_3_), 24.3 (C5′-OC(OMe)*Me*_2_), 25.6 (Si-C*Me*_3_), 41.2 (C2′), 48.6 (C5′-OC(O*Me*)Me_2_), 60.3 (C5′), 72.2 (C3′), 84.7 (C4′), 86.2 (C1′), 100.2 (C5′-O*C*(OMe)Me_2_), 110.6 (C5),135.3 (C6), 150.6 (C2), 164.2 (C4). High resolution positive ion ESI-MS: *m/z* obsd. 451.2241 [M+Na]^+^; calcd. 451.2240 [M+Na]^+^. 

*N^4^-Benzoyl-5′-O*-*(1-methoxy-1-methylethyl)-3′-*O*-(*tert*-butyldimethylsilyl)-2′-deoxycytidine* (**3b**) was obtained in 91% yield (1.06 mmol, 0.55 g) from compd. **2b**, as described above for compd. **3a**. ^1^H-NMR (400 MHz, CDCl_3_): δ = −0.07 (s, 6H, Si*Me*_2_), 0.82 (s, 9H, SiC*Me*_3_), 1.33 and 1.35 (s, 2 × 3H, OC(OMe)*Me*_2_), 2.11–2.17 (m, 1H, H2′), 2.43–2.50 (m, 1H, H2′), 3.17 (s, 3H, OC(O*Me*)Me_2_), 3.53 (dd, *J* = 10.9 and 2.6 Hz, 1H, H5′), 3.70 (dd, *J* = 10.9 and 3.0, 1H, H5′′), 3.97–4.01 (m, 1H, H4′), 4.30–4.34 (m, 1H, H3′), 6.17–6.20 (m, 1H, H1′), 7.44 (t, *J* = 7.7 Hz, 3H, Bz&H5), 7.53 (t, *J* = 7.7 Hz, 1H, Bz), 7.85 (d, *J* = 7.7 Hz, 2H, Bz); 8.45 (d, *J* = 7.5 Hz, 1H, H6), 9.22 (br s, 1H, NH); ^13^C-NMR (101 MHz, CDCl_3_): δ = −4.9 and −4.6 (Si*Me*_2_), 17.9 (Si-*C*Me_3_), 24.3 (C5′-OC(OMe)*Me*_2_), 25.6 (Si-C*Me*_3_), 42.1 (C2′), 48.7 (C5′-OC(O*Me*)Me_2_), 59.1 (C5′), 70.3 (C3′), 86.3 (C4′), 86.9 (C1′), 96.0 (C5), 100.3 (C5′-O*C*(OMe)Me_2_), 127.7 (Bz), 128.8 (Bz), 133.0 (Bz), 133.2 (Bz), 144.7 (C6), 154.7 (C2), 162.3 (C4), 167.0 (Bz). High resolution negative ion ESI-MS: *m/z* obsd. 516.2564 [M−H]^−^; calcd. 516.2530 [M−H]^−^. 

*N^6^-Benzoyl-5′-O-(1-methoxy-1-methylethyl)-3′-O-(*tert*-butyldimethylsilyl)-2′-deoxyadenosine* (**3c**) was obtained in 81% yield (1.07 mmol, 0.58 g) from compd. **2c**, as described above for compd. **3a**. ^1^H-NMR (500 MHz, CD_3_CN): δ = 0.00 (s, 6H, Si*Me*_2_), 0.80 (s, 9H, SiC*Me*_3_), 1.14 (s, 6H, OC(OMe)*Me*_2_, 2.27–2.37 (m, 1H, H2′), 2.67–2.73 (m, 1H, H2′), 2.92 (s, 3H, OC(O*Me*)Me_2_), 3.36–4.03 (m, 3H, H4′&2 × H5′), 4.56–4.62 (m, 1H, H3′), 6.32 (br s, 1H, H1′), 7.37 (t, *J* = 7.7 Hz, 2H, Bz), 7.47 (t, *J* = 7.7 Hz, 1H, Bz), 7.86 (d, *J* = 7.7 Hz, 2H, Bz), 8.24 (s, 1H, H2), 8.50 (s, 1H, H8), 9.44 (s, 1H, NH); ^13^C-NMR (126 MHz, CD_3_CN): δ = −6.0 (Si*Me*_2_), 17.3 (Si-*C*Me_3_), 23.2 and 23.4 (C5′-OC(OMe)*Me*_2_), 24.8 (Si-C*Me*_3_), 39.7 (C2′), 47.5 (C5′-OC(O*Me*)Me_2_), 60.0 (C5′), 72.0 (C3′), 83.9 (C4′), 86.1 (C1′), 99.6 (C5′-O*C*(OMe)Me_2_), 124.2 (C5), 127.8 (Bz), 128.2 (Bz), 132.1 (Bz), 133.5 (Bz), 141.9 (C8), 149.4 (C4), 151.5 (C2&C6), 164.9 (Bz). Hugh resolution negative ion ESI-MS: *m/z* obsd. 540.2663 [M−H]^−^, calcd. 540.2642 [M−H]^−^. 

*5′-O-(1-Methoxy-1-methylethyl)thymidine* (**4a**). Compound **3a** (0.38 mmol, 165 mg) was treated with 2 equiv. of tetrabutylammonium fluoride (0.75 mmol, 0.197 g) in THF (4 mL). After stirring for 4.5 h at r.t., the mixture was equilibrated between EtOAc and aq. NaHCO_3_, the organic phase was evaporated to dryness and the residue was purified by silica gel column chromatography using DCM containing 1-5% MeOH as eluent. Yield 91% (111 mg). ^1^H-NMR (500 MHz, CDCl_3_): δ = 1.39 and 1.41 (2 × s, 2 × 3H, C5′-OC(OMe)*Me*_2_), 1.93 (s, 3H, C5-Me), 2.14–2.19 (m, 1H, H2′), 2.38–2.41 (m, 1H, H2′), 3.23 (s, 3H, C5′-OC(O*Me*)Me_2_), 3.64 (d, *J* = 10.6 Hz, 1H, H5′), 3.69 (d, *J* = 10.6 Hz, 1H, H5′), 4.15 (s, 1H, H4′), 4.48 (m, 1H, H3′), 6.41 (dd, *J* = 6.3 and 6.3 Hz, 1H, H1′), 7.61 (s, 1H, H6), 9.50 (br s, 1H NH); ^13^C-NMR (126 MHz, CDCl_3_): δ = 12.3 (C5-Me), 24.2 (C5′-OC(OMe)*Me*_2_), 40.7 (C2′), 48.6 (C5′-OC(O*Me*)Me_2_), 60.8 (C5′), 72.2 (C3′), 84.8 (C4′), 85.8 (C1′), 100.2 (C5′-O*C*(OMe)Me_2_), 110.6 (C5), 135.6 (C6), 150.4 (C2), 163.8 (C4). High resolution negative ion ESI-MS: *m/z* obsd. 313.1424 [M−H]^−^, calcd. 313.1400 [M−H]^−^.

*N^4^-Benzoyl-5′-O-(1-methoxy-1-methylethyl)-2′-deoxycytidine* (**4b**) was obtained in 81% yield (0.87 mmol, 0.35 g) from **3b** as described above for compd. **4a**. ^1^H-NMR (400 MHz, CDCl_3_): δ = 1.28 and 1.31 (2 × s, 2 × 3H, C5′-OC(OMe)*Me*_2_), 2.21–2.28 (m, 1H, H2′), 2.65–2.73 (m, 1H, H2′), 3.23 (s, 3H, C5′-OC(O*Me*)Me_2_), 3.67 (dd, *J* = 10.9 and 2.9 Hz, 1H, H5′), 3.76 (dd, *J* = 3.0 Hz, 1H, H5′′), 4.20–4.24 (m, 1H, H4′), 4.46–4.49 (m, 1H, H3′), 4.30–4.41 (m, 1H, H3′), 6.31–6.34 (m, 1H, H1′), 7.40–7.56 (m, 3H, Bz&H5), 7.61 (t, *J* = 7.4 Hz, 1H, Bz) 7.93 (d, *J* = 7.4 Hz, 2H, Bz), 8.48 (d, *J* = 7.5 Hz, 1H, H6); 9.60 (s, 1H NH); ^13^C-NMR (101 MHz, CDCl_3_): δ = 24.3 (C5′-OC(OMe)*Me*_2_), 42.1 (C2′), 48.8 (C5′-OC(O*Me*)Me_2_), 60.1 (C5′), 70.6 (C3′), 86.4 (C4′), 87.3 (C1′), 98.2 (C5), 100.3 (C5′-O*C*(OMe)Me_2_), 127.7 (Bz), 128.9 (Bz), 133.1 (Bz), 133.6 (Bz), 145.0 (C6), 155.1 (C2), 162.4 (C4), 167.0 (Bz). High resolution negative ion ESI-MS: *m/z* obsd. 402.1692 [M−H]^−^; calcd. 402.1665 [M−H]^−^.

*N^6^-Benzoyl-5′-O-(1-methoxy-1-methylethyl)-2′-deoxyadenosine* (**4c**) was obtained in 89% yield (0.94 mmol, 0.40 g) from **3c** as described above for compd. **4a**. ^1^H-NMR (500 MHz, CD_3_CN): δ = 1.26 (2s, 6H, C5′-OC(OMe)*Me*_2_), 2.40–2.50 (m, 1H, H2′), 2.70–2.79 (m, 1H, H2′), 3.04 (s, 3H, C5′-OC(O*Me*)Me_2_), 3.40–4.10 (m, 3H, H4′&2 × H5′), 4.56 (m, 1H, H3′), 6.45–6.48 (m, 1H, H1′), 7.50 (t, *J* = 7.6 Hz, 2H, Bz), 7.60 (t, *J* = 7.6 Hz, 1H, Bz), 7.99 (d, *J* = 7.6 Hz), 8.42 (s, 1H, H2), 8.63 (s, 1H, H8); 9.69 (s, 1H, NH); ^13^C-NMR (126 MHz, CD_3_CN): δ = 23.3 and 23.5 (C5′-OC(OMe)*Me*_2_), 39.6 (C2′), 47.5 (C5′-OC(O*Me*)Me_2_), 60.3 (C5′), 70.8 (C3′), 83.8 (C4′), 85.9 (C1′), 100.3 (C5′-O*C*(OMe)Me_2_), 124.0 (C5), 127.8 (Bz), 128.3 (Bz), 132.2 (Bz), 133.2 (Bz), 141.8 (C8 ), 149.4 (C4), 151.5 (C2), 151.6 (C6), 165.1 (Bz). High resolution negative ion ESI-MS: *m/z* obsd. 426.1783 [M−H]^−^; calcd. 426.1777 [M−H]^−^. 

*5′-O-(1-Methoxy-1-methylethyl)thymidine 3′-(2-cyanoethyl-N,N-diisopropyl)phosphoramidite* (**5a**). Glassware and stirrer bar were dried for 15 hours in an oven (120 °C). All chemicals and reagents were placed in a box under nitrogen. Compd. **4a** (4.27 mmol, 0.40 g) was dissolved in dry DCM (20 mL). Triethylamine (5 equiv., 6.37 mmol, 0.885 mL) and 1-chloro-1-(2-cyanoethoxy)-*N*,*N*-diisopropylphosphinamine (1.1 equiv., 0.330 mL) were added and the mixture was stirred for 2.5 h at r.t. under nitrogen. The mixture was then loaded onto a silica gel column and the product was eluted with a 50:49:1 mixture of EtOAc, petroleum ether and Et_3_N. Compd. **5a** was obtained as a white foam (yield 85%), which was stored at −18 °C. For the mixture of *R*_P_ and *S*_P_ diastereomers: ^1^H-NMR (500 MHz, CD_3_CN): δ = 1.17 (d, *J* = 6.8 Hz, 12H, iPr), 1.33 and 1.34 (2 × s, 6H, C5′-OC(OMe)*Me*_2_), 1.84 (s, 3H, C5-Me), 2.20–2.45 (m, 2H, 2 × H2′), 2.67 (t, *J* = 5.9 Hz, 2H, OCH_2_C*H*_2_CN), 3.15 and 3.17 (2 × s, 3H, C5′-OC(O*Me*)Me_2_), 3.53–4.25 (m, 7H, 2 × H5′& H4′&OC*H*_2_CH_2_CN&2 × NC*H*Me_2_), 4.48–4.59 (m, 1H, H3′), 6.24 (m, 1H, H1′), 7.52 and 7.54 (2 × s, 1H, H6), 9.80 (br s, 1H, NH); ^13^C-NMR (126 MHz, CD_3_CN): δ = 11.3 (C5-Me), 19.7 (OCH_2_*C*H_2_CN), 23.4 (C5′-OC(OMe)*Me*_2_), 23.6 (NCH*Me*_2_), 38.7 (C2′), 42.6 (C5′-OC(O*Me*)Me_2_), 47.8 (N*C*HMe_2_), 57.9 (O*C*H_2_CH_2_CN), 60.2 (C5′), 73.4 (C3′), 81.7 (C4′), 84.1 (C1′), 99.8 (C5′-O*C*(OMe)Me_2_), 109.9 (C5), 118.1 (OCH_2_CH_2_*C*N), 135.6 (C6), 150.6 (C2), 163.8 (C4); ^31^P-NMR (CD_3_CN): δ = 149.3 and 148.9. Positive ion ESI-MS: *m/z* obsd. 537.25 [M+Na]^+^; calcd. 537.25 [M+Na]^+^.

*N^4^-Benzoyl-5′-O-(1-methoxy-1-methylethyl)-2′-deoxycytidine 3′-(2-cyanoethyl-N,N-diisopropyl)-phosphoramidite* (**5b**) was obtained in 86% yield (0.75 mmol, 0.45 g) from **4b** as described above for compd. **5a**. For the mixture of *R*_P_ and *S*_P_ diastereomers: ^1^H-NMR (500 MHz, CDCl_3_): δ = 1.20 (d, *J* = 6.6 Hz, 12H, iPr), 1.40 (2 × s, 6H, C5′-OC(OMe)*Me*_2_), 2.20–2.45 (m, 2H, 2 × H2′), 2.64 (t,*J* = 5.9 Hz, 2H, OCH_2_C*H*_2_CN), 3.22 (2 × s, 3H, C5′-OC(O*Me*)Me_2_), 3.45–4.45 (m, 7H, 2 × H5′&H4′&OC*H*_2_CH_2_CN&2 × NC*H*Me_2_), 4.51–4.60 (m, 1H, H3′), 6.25–6.35 (m, 1H, H1′), 7.46 (m, 3H, Bz&H5), 7.57 (m, 1H, Bz), 7.95 (m, 2H, Bz), 8.42 (m, 1H, H6), 9.60 (br s, 1H, NH); ^13^C-NMR (126 MHz, CDCl_3_): 18.5 (OCH_2_*C*H_2_CN), 22.4 (C5′-OC(OMe)*Me*_2_), 22.6 (NCH*Me*_2_), 38.9 (C2′), 41.4 (C5′-OC(O*Me*)Me_2_), 47.0 (N*C*HMe_2_), 57.0 (O*C*H_2_CH_2_CN), 58.2 (C5′), 71.1 (C3′), 85.2 (C4′), 86.0 (C1′), 95.0 (C5), 98.5 (C5′-O*C*(OMe)Me_2_), 116.2 (OCH_2_CH_2_*C*N), 126.2 (Bz), 127.2 (Bz), 131.3 (Bz), 131.4 (Bz), 143.3 (C6), 152.8 (C2), 156.6 (C4), 150.8 (C2), 165.5 (Bz); ^31^P-NMR (CDCl_3_): δ = 148.5 and 149.0. Negative ion ESI-MS: *m/z* obsd. 602.28 [M−H]^−^; calcd. 602.27 [M−H]^−^.

*N^6^-Benzoyl-5′-O-(1-methoxy-1-methylethyl)-2′-deoxyadenosine 3′-(2-cyanoethyl-N,N-diisopropyl)-phosphoramidite* (**5c**) was obtained in 83% yield (0.70 mmol, 0.44 g) from **4c **as described above for compd. **5a**. For the mixture of *R*_P_ and *S*_P_ diastereomers: ^1^H-NMR (500 MHz, CDCl_3_): δ = 1.18 (d, *J* = 6.6 Hz, 12H, iPr), 1.39 and 1.41 (2 × s, 6H, C5′-OC(OMe)*Me*_2_), 2.20–2.45 (m, 2H, 2 × H2′), 2.66 (t,*J* = 5.9 Hz, 2H, OCH_2_C*H*_2_CN), 3.22 and 3.24 (2 × s, 3H, C5′-OC(O*Me*)Me_2_), 3.43–4.25 (m, 7H, 2 × H5′&H4′&OC*H*_2_CH_2_CN&2 × NC*H*Me_2_), 4.53–4.59 (m, 1H, H3′), 6.28 (m, 1H, H1′), 7.46 (m, 2H, Bz), 7.58 (m, 1H, Bz), 7.96 (m, 2H, Bz), 8.28 (s, 1H, H2), 8.46 (s, 1H, H8), 9.30 (br s, 1H, NH); ^13^C-NMR (126 MHz, CDCl_3_): 18.6 (OCH_2_*C*H_2_CN), 22.5 (C5′-OC(OMe)*Me*_2_), 22.7 (NCH*Me*_2_), 39.3 (C2′), 41.4 (C5′-OC(O*Me*)Me_2_), 47.0 (N*C*HMe_2_), 56.9 (O*C*H_2_CH_2_CN), 58.0 (C5′), 70.9 (C3′), 83.9 (C4′), 85.3 (C1′), 98.5 (C5′-O*C*(OMe)Me_2_), 115.9 (OCH_2_CH_2_*C*N), 122.0 (C5), 126.1 (Bz), 127.0 (Bz), 131.1 (Bz), 131.4 (Bz), 142.8 (C8), 148.2 (C4), 150.2 (C6), 150.8 (C2), 163.8 (Bz); ^31^P-NMR (CDCl_3_): δ = 148.5 and 148.9. Negative ion ESI-MS: *m/z* obsd. 626.30 [M−H]^−^, calcd. 626.29 [M−H]^−^.

*5′-O-(1-Methoxy-1-methylethyl)-3′-O-(hex-5-ynoyl)thymidine* (**6**). Dicyclohexylcarbodiimide (0.722 mmol, 0.149 g) was dissolved in dry dioxane (2 mL) and the solution obtained was added dropwise on an ice-bath to 5-hexynoic acid (1.44 mmol, 0.162 g, 0.160 mL) in dioxane (1 mL). After stirring for 2 h at r.t., the precipitated dicyclohexylurea was filtrated off and the volatiles were removed under reduced pressure, giving hex-5-ynoic anhydride as a slightly yellow liquid. Compd. **4a** (0.35 mmol, 0.111 g) was dissolved in dry pyridine (5 mL), hex-5-ynoic anhydride and a catalytic amount of dimethylaminopyridine were added, and the reaction was allowed to proceed 17 h at rt. The mixture was concentrated under reduced pressure and the residue was purified by silica gel chromatography applying gradient elution with 1–5% MeOH in DCM containing 1% triethylamine. Compound **6** was obtained as white foam in 92% yield (0.32 mmol, 0.132 g). ^1^H-NMR (500 MHz, CDCl_3_): δ = 1.41 and 1.42 (2 × s, 2 × 3H, C5′-OC(OMe)*Me*_2_), 1.84–1.89 (m, 2H, H3 of hex-5-ynoyl), 1.95 (s, 3H, C5-Me), 2.02–2.04 (m, 1H, H2′), 2.18–2.24 (m, 1H, H2′), 2.28–2.31 (m, 2H, H2 of hex-5-ynoyl), 2.39–2.43 (m, 2H, H4 of hex-5-ynoyl), 2.52 (t, *J* = 7.4 Hz, 1H, H6 of hex-5-ynoyl), 3.25 (s, 3H, C5′-OC(O*Me*)Me_2_), 3.70–3.99 (m, 2H, H5′), 4.21 (m, 1H, H4′), 5.30–5.32 (m, 1H, H3′), 6.43 (dd, *J* = 6.3 Hz, 1H, H1′), 7.62 (s, 1H, H6), 9.50 (s, 1H NH); ^13^C-NMR (126 MHz, CDCl_3_): δ = 12.5 (C5-*Me*), 17.7 (C4 of hex-5-ynoyl), 23.3 (C3 of hex-5-ynoyl), 24.4 (C5′-OC(OMe)*Me*_2_), 32.7 (C2 of hex-5-ynoyl), 37.9 (C2’), 48.8 (C5′-OC(O*Me*)Me_2_), 61.1 (C5′), 69.5 (C6 of hex-5-ynoyl), 75.4 (C3′), 82.9 (C5 of hex-5-ynoyl), 83.9 (C4′), 84.6 (C1′), 100.4 (C5′-O*C*(OMe)Me_2_), 111.2 (C5), 135.2 (C6), 150.8 (C2), 164.0 (C4), 172.7 (C=O of hex-5-ynoyl). High resolution negative ion ESI-MS: *m/z* obsd. 407.1841 [M−H]^−^, calcd. 407.1818 [M−H]^−^.

*5′-O-(4,4′-Dimethoxytrityl)-3′-O-(hex-5-ynoyl)thymidine* (**7**). Thymidine was converted to 5′-*O*-(4,4′-dimethoxytrityl)thymidine as described in literature [30] and acylated with hex-5-ynoic anhydride as described above for compound **6**. The yield of acylation was 99%. ^1^H- and ^13^C-NMR spectroscopic data was identical with that given by Oyelere *et al.* [31]. High resolution negative ion ESI-MS: *m/z* obsd. 637.2534 [M−H]^−^, calcd. 637.2550 [M−H]^−^.

*6-O-(*p*-Toluenesulfonyl)-β-cyclodextrin* (**8**). Monotosylated β-cyclodextrin was prepared by the method of Kuzuya *et al*. [19] The ^1^H- and ^13^C-NMR spectra of the product were identical to those reported previously in literature [32]. Negative ion ESI-MS: *m/z* obsd 1287.41 [M−H]^−^, calcd. 1287.37 [M−H]^−^.

*6-Azido-6-deoxy-β-cyclodextrin* (**9**) was obtained by displacing the *p*-toluenesulfonyloxy group from compd. **8** with azide ion as reported by Petter *et al.* [32]. The ^1^H- and ^13^C-NMR chemical shifts were identical with those reported previously [32]. Negative ion ESI-MS: *m/z* obsd. 1158.36 [M−H]^−^, calcd. 1158.37 [M−H]^−^.

*Peracetylated 6-azido-6-deoxy-β-cyclodextrin* (**10a**). The azido-functionalized β-cyclodextrin (**9**) was peracetylated with acetic anhydride in pyridine at r.t., using 4 quiv. of acetic anhydride compared to the amount of free OH groups. The product was purified by silica gel chromatography applying a stepwise gradient elution with 1–5% MeOH in DCM containing 1% Et_3_N. The yield was 73%. ^1^H-NMR (500 MHz, DMSO-*d*_6_) 2.05 (br s, 60H, Ac), 3.65 (d, *J* = 10 Hz, 1H, C*H*HN_3_), 3.77 (d, *J* = 10 Hz, CH*H*N_3_), 3.85–3.90 (br s, 7H, H4), 4.03–4.12 (m, 7H, H5), 4,18–4.28 (m, 6H, H6b), 4.40–4.50 (m, 6H, H6a), 4.65–4.75 (m, 7H, H2), 5.00–5.15 (m, 7H, H3), 5.18–5.23 (m, 7H, H1); ^13^C-NMR (126 MHz, DMSO-*d*_6_): 169.5 (Ac), 97.2 (C1), 77.3 (C4), 71.0 (C5), 70.8 (C3), 70.0 (C2), 62.6 (C6), 50.8 (*C*H_2_N_3_), 21.0 (Ac). Positive ion ESI-MS: *m/z* obsd. 2022.56 [M+Na]^+^, calcd. 2022.58 [M+Na]^+^.

*Permethylated 6-azido-6-deoxy-β-cyclodextrin* (**10b**). Permethylation of the azido-functionalized β-cyclodextrin was achieved by treating **9** over a weekend with a modest excess of methyl iodide (1.25 equiv.) and sodium hydroxide (1.25 equiv.) compared to the amount of free hydroxyl functions, as described previously by Schomburg *et al.* [20,21]. The yield was 91%. The ^1^H-NMR spectrum was identical with that reported earlier [33]. ^13^C-NMR (126 MHz, DMSO-*d*_6_): 96.8 (C1), 80.6 (C3), 80.2 (C2), 78.4 (C4), 70.2 (*C*5-CH_2_N_3_), 69.4 (C6-N_3_), 59.8 (O6-Me), 57.0 (O2-Me&O3-Me), 50.4 (*C*H_2_N_3_). Negative ion ESI-MS: *m/z* obsd. 1474.76 [M+Cl]^−^, calcd. 1475.66 [M+Cl]^−^.

*Immobilization of 3′-*O*-(hex-5-ynoyl)thymidines to cyclodextrin supports.* 3′-*O*-(hex-5-ynoyl)thymidines **6** and **7** were immobilized to the azido-functionalized supports **10a**,**b** by Cu^+^-promoted 1,3-dipolar cyclo addition. Accordingly, the appropriate nucleoside, either **6** (3.03 mmol, 1.25 g) or **7** (1.03 mmol, 0.66 g) in dioxane (15 mL) was mixed with either **10a** or **10b** in the same solvent. Aq. CuSO_4_ (50 mmol·L^−1^) and sodium ascorbate (0.1 mmol·L^−1^) were added and the reaction was allowed to proceed for 3 d at r.t. Aq. CuSO_4_.5H_2_O and aq. sodium ascorbate were added daily to ensure quantitative formation of the 1,2,3-triazole linkages. The products **11a**–**c** were obtained in about 50% yield as white foams by silica gel chromatography applying first a stepwise gradient from 4% to 34% cyclohexane in EtOAc containing 1% triethylamine, and then a stepwise gradient of 5% to 10% MeOH in DCM containing 1% triethylamine. The identity and homogeneity of the products were verified by ESI-MS and HPLC, respectively. 

### 3.3. Oligonucleotide Synthesis

#### 3.3.1. Chain Assembly from 5′-*O*-(4,4′-Dimethoxytrityl) Protected Nucleoside Phosphoramidites

Support **11b** (0.180 g, 68.2 μmol) was dissolved in DCM containing 3% dichloroacetic acid (5 mL) and stirred for 1 h. The mixture was diluted with DCM (25 mL) and washed with saturated aq. sodium bicarbonate (3 × 10 mL). Combined aqueous phases were back-extracted with DCM (20 mL). The combined organic phases were dried over anhydrous sodium sulfate and evaporated to dryness, giving 0.177 g deprotected material that still contained the DMTr alcohol. The product was subjected to coupling without purification. 

Deprotected **11b** (0.177 g) and commercially available 5′-*O*-DMTr-thymidine 3′-(2-cyanoethyl-*N,N*-diisopropylphosphoramidite (1.5 equiv., 100.6 μmol, 0.075 g) were dissolved in anhydrous MeCN (0.775 mL) under nitrogen and 0.45 mol·L^−1^ solution of tetrazole in MeCN (1.5 equiv., 0.225 mL) was added. The mixture was stirred under nitrogen for 1 h, iodine oxidation solution (I_2_:H_2_O:THF:2,6-lutidine 0.43 g:4 mL:8 mL:2 mL; 1.2 equiv., 0.820 mL) was added and stirred for additional 30 min. The mixture was concentrated under reduced pressure, the residue was dissolved in DCM (20 mL) and washed with saturated aq sodium bisulfite (3 × 10 mL) and brine (3 × 10 mL). Combined aqueous phases were back-extracted with DCM (20 mL). The organic phases were combined, dried over anhydrous sodium sulfate and evaporated to dryness to give 0.196 g of white foam. The product was used for the next coupling cycle without purification. According to UV-spectrophotometric assay, the trimer was obtained in 85% yield, which corresponds to 92% coupling yield. The homogeneity of product was verified by RP-HPLC (see Figure 2A). 

#### 3.3.2. Chain Assembly from 5′-*O*-(1-Methoxy-1-methylethyl) Protected Nucleoside Phosphoramidites

Glassware and stirrer bar were dried in the oven (120 °C). All chemicals and reagents were placed in a box under Nitrogen atmosphere. The solvents were dried over 3 Å molecular sieves.

Acetylated and methylated cyclodextrin supports **11a** and **11c** were treated with a mixture of MeOH (2 mL) and 0.1 mol·L^−1^ HCl in 1,4-dioxane (6 mL) for 30 min at r.t. The volatiles were removed under reduced pressure and traces of MeOH were removed by coevaporation with dry pyridine and MeCN. Support **11c** was additionally purified chromatographically to remove the 4,4′-dimethoxytrityl alcohol released. Deprotected support **11a** was used to assemble 3′-TTT-5′ and support **11c** to assemble 3′-TTT-5′ and 3′-TTCAT-5′. 

The deprotected support was dissolved in MeCN (2mL) and the desired 5′-acetal protected building block (**5a**–**c**) in MeCN (1.5 equiv. in 1 mL) and 4,5-dicyanoimidazole (1.5 equiv. in 0.52 mL MeCN) were added under nitrogen. The mixture was stirred for 6 h and the oxidation solution (1.1 mL containing 1.5 equiv. of iodine in a 2:4:1 mixture of water, THF and 2,6-lutidine) was added. After 30 min stirring, the mixture was diluted with DCM (20 mL) and washed with an equal volume of aq. NaHSO_3_ to remove the traces of iodine. The organic phase was dried over Na_2_SO_4_, filtered and evaporated to dryness under reduced pressure. The residue that still contained traces of 2,6-lutidine, was dissolved in MeOH (2 mL) and a sufficient volume of 0.1 mol L^−1^ HCl in dioxane was added to neutralize lutidine and adjust pH slightly below 4. After 30 min at r.t., the mixture was evaporated to dryness and the residue was coevaporated with pyridine and MeCN, and subjected to flash chromatographic separation with 1–15% MeOH in DCM to remove the remnants of the first coupling reaction. The subsequent couplings were then carried out in a similar manner, except that upon the two last couplings the support was dissolved in a 4:1 (*v/v*) mixture of MeCN and DCM instead of MeCN. The identitiy of the product was checked by negative ion ESI-MS after each coupling and deprotection.

On assembling the 3′-TTT-5′ trimer on the acetylated support **11a**, the negative ion ESI-MS values after each coupling and deprotection were (calculated values in parentheses): CD-T 2334.7 (2334.7) [M−H]^−^, CD-TT 2691.7 (2691.8) [M−H]^−^, CD-TTT 3048.8 (3048.9) [M−H]^−^. The spectrophotometrically determined yield was 87%, corresponding to 93% average coupling yield. On assembling the same trimer on the methylated support **11c**, the negative ion ESI -MS *m/z* values for the support-bound phosphate protected trimer were 2489.0 (calcd. 2489.0). In both cases, the *m/z* value for the released and deprotected 3′-TTT-5′ was 849.15 (calcd. 849.17). The homogeneity of the products was verified by RP-HPLC (see Figure 2 B and C). 

On preparing the pentamer on methylated cyclodextrin support **11c** (96 μmol, 170 mg), the identity of the product was checked by negative ion ESI-MS after each coupling and deprotection (calculated values in parentheses): CD-T 1774.8 (1774.8) [M−H]^−^, CD-TT 2131.8 (2131.9) [M−H]^−^, CD-TTC 2578.1 (2578.0) [M−H]^−^, CD-TTCA 1523.6 (1523.5) [M−2H]^2−^, CD-TTCAT 1702.0 (1702.1) [M−2H]^2−^. The overall yields after each coupling and subsequent removal of the 5′-protecting group were (CD refers to the cyclodextrin support): CD-TT-5′ (88%, 84 μmol, 180 mg), CD-TTC-5′ (77%, 74 μmol, 190 mg), CD-TTCA-5′ (64%, 61 μmol, 186 mg) and CD-TTCAT-5′ (52%, 50 μmol, 170 mg). The identity of the released and deprotected pentamer was verified by negative ion ESI^−^-MS: *m/z* obsd. 1451.31 [M−H]^−^, calcd. 1451.28 [M−H]^−^. The homogeneity of the product was verified by reversed phase HPLC (see Figure 4). The mass of the crude product (after removal of the support by extraction) was 64 mg and the isolated yield (after HPLC purification) 52 mg (37% of theoretical).

## 4. Conclusions

A useful method for the preparation of short oligodeoxyribonucleotides in solution has been developed. The essential features of the method include use of 1-methoxy-1-methylethyl group as the 5′-*O* protecting group and permethylated β-cyclodextrin as a soluble support. Acid-catalyzed methanolysis of this 5′-acetal protection gives easily removable volatile products and hydrophobic cyclodextrin support allows efficient coupling on using only 50% excess of the monomeric building block, rapid flash chromatographic purification after each coupling cycle and extractive removal of the support after ammonolytic release/deprotection of the oligonucleotide. 
